# Coronavirus disease 2019 (COVID-19) outbreak on an in-patient medical unit associated with unrecognized exposures in common areas—Epidemiological and whole-genome sequencing investigation

**DOI:** 10.1017/ice.2023.34

**Published:** 2023-11

**Authors:** Dylan C. Kain, Sandra Isabel, Mariana Abdulnoor, Karel Boissinot, Richard De Borja, Amanda Filkin, Bernard Lam, Jason Li, Ilinca Lungu, Liz McCreight, Allison McGeer, Tony Mazzulli, Aimee Paterson, Philip Zuzarte, Felicia Vincelli, Cassandra Bergwerff, Ramzi Fattouh, Jared T. Simpson, Jennie Johnstone

**Affiliations:** 1 Department of Medicine, University of Toronto, Toronto, Ontario, Canada; 2 Department of Laboratory Medicine and Pathobiology, University of Toronto, Toronto, Ontario, Canada; 3 Sick Kids Hospital, Department of Infectious Disease, Toronto, Ontario, Canada; 4 Department of Laboratory Medicine, St. Michael’s Hospital, Unity Health Toronto, Toronto, Ontario, Canada; 5 Ontario Institute for Cancer Research, Toronto, Ontario, Canada; 6 Occupational Health, Sinai Health, Toronto, Ontario, Canada; 7 Infection Prevention and Control, Sinai Health, Toronto, Ontario, Canada; 8 Lunenfeld-Tanenbaum Research Institute, Sinai Health System, Toronto, Ontario, Canada; 9 Department of Microbiology, Sinai Health System/University Health Network, Toronto, Ontario, Canada; 10 Li Ka Shing Knowledge Institute, Unity Health Toronto, Toronto, Ontario, Canada; 11 Department of Molecular Genetics, University of Toronto, Toronto, Ontario, Canada; 12 Dalla Lana School of Public Health, University of Toronto, Toronto, Ontario, Canada

## Abstract

**Objective:**

Severe acute respiratory syndrome coronavirus 2 (SARS-CoV-2) hospital outbreaks have been common and devastating during the coronavirus disease 2019 (COVID-19) pandemic. Understanding SARS-CoV-2 transmission in these environments is critical for preventing and managing outbreaks.

**Design:**

Outbreak investigation through epidemiological mapping and whole-genome sequencing phylogeny.

**Setting:**

Hospital in-patient medical unit outbreak in Toronto, Canada, from November 2020 to January 2021.

**Participants:**

The outbreak involved 8 patients and 10 staff and was associated with 3 patient deaths.

**Results:**

Patients being cared for in geriatric chairs at the nursing station were at high risk for both acquiring and transmitting SARS-CoV-2 to other patients and staff. Furthermore, given the informal nature of these transmissions, they were not initially recognized, which led to further transmission and missing the opportunity for preventative COVID-19 therapies.

**Conclusions:**

During outbreak prevention and management, the risk of informal patient care settings, such as geriatric chairs, should be considered. During high-risk periods or during outbreaks, efforts should be made to care for patients in their rooms when possible.

Since its emergence, severe acute respiratory syndrome coronavirus 2 (SARS-CoV-2) has rapidly spread worldwide, and healthcare facilities have been a common setting for coronavirus disease 2019 (COVID-19) outbreaks. Understanding the mechanisms of transmission in hospital outbreaks is critical to preventing and mitigating risks to patients and staff.

Traditional epidemiologic outbreak investigations solve transmission dynamics in certain cases but are hampered when SARS-CoV-2 community incidence is high. Whole-genome sequencing (WGS) has demonstrated that presumed “superspreading” events were actually multiple separate introductions,^1,2^ and an assumed international introduction of a SARS-CoV-2 variant into a long-term care (LTC) facility was in fact acquired locally.^3^ The benefits of WGS for outbreak management are increasingly acknowledged by infection and prevention and public health professionals.^4^ We describe a COVID-19 outbreak in a hospital in-patient unit with traditional epidemiological and WGS investigations to pinpoint critical transmission risk events and to prevent their recurrence.

## Methods

### Outbreak investigation

A SARS-CoV-2 outbreak occurred on a general medicine ward, in Toronto, Canada, from November 22, 2020, to January 4, 2021 (prior to widespread vaccination). We performed a retrospective chart review of all patients on the unit. Electronic room tracking was used to identify shared rooms. Nursing notes were reviewed to analyze patient location and transit in the hospital (ie, room, transportation for test, observation near the nursing station) and COVID-19–related symptoms.^5^ We obtained staff symptoms, onset date, and SARS-CoV-2 test results through an outbreak line list. Data regarding other staff interactions and exposures were collected through individual interviews. Patient rooms had 6 air exchanges per hour and the nursing station had 10 air exchanges per hour.

### PCR and WGS

SARS-CoV-2 rRT-PCR testing was performed on nasopharyngeal swabs using the ALLPLEX 2019-nCoV Assay (Seegene, Toronto, Canada) or NxTAG Respiratory Pathogen Panel Assay (Luminex, Toronto, Canada) in our institution. Nucleic acids for all 20 SARS-CoV-2–positive samples retrieved for WGS were extracted using the NucliSENS easyMag platform (bioMerieux, Montreal, Québec). Two patient samples were no longer available. Next-generation sequencing was performed using the MinION instrument with R9.4.1 flow cells (Oxford Nanopore Technologies, UK). ARTIC Network Pipeline version 1.1.3 software with the v3 amplicon set was used with Nanopolish to determine consensus genome sequences.^6^

### Phylogenetic analyses

**Fig. 1.**
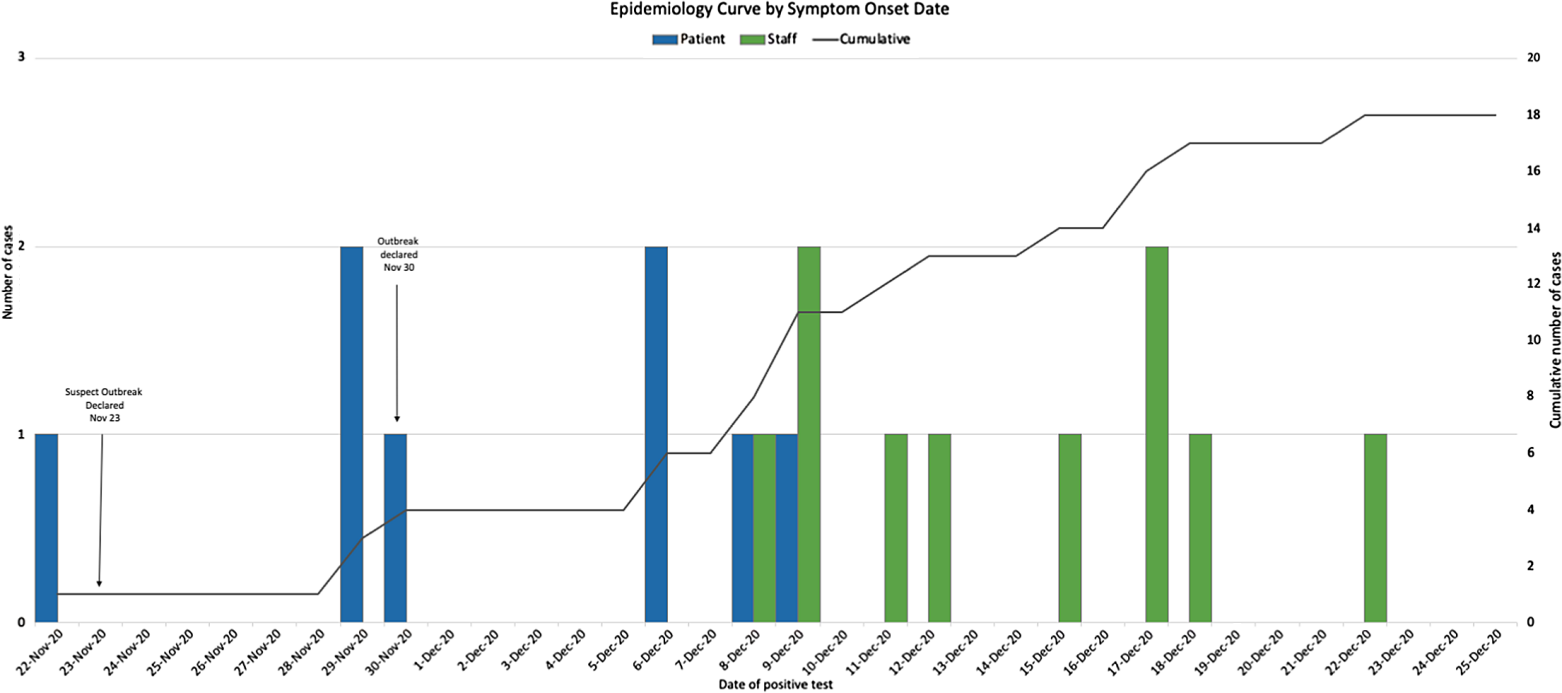
Epidemiology curve of patient and staff cases by symptom-onset date. A suspected outbreak and an outbreak were declared November 23 and 30, 2020, respectively. Blue and green bars represent patient and staff cases, respectively. Cumulative cases are shown on the right axis.

In total, 41,712 SARS-CoV-2 genome sequences from Canada were obtained from GISAID^7^ on May 22, 2021 (Supplementary Table 1 online). Maximum likelihood tree using substitution model HKY and 1,000 bootstrap analyses was created in IQ-TREE verison 1.6.2 software.^8,9^ The phylogenetic tree was visualized and annotated in iTOL version 6.5.2 software.^10^ We selected the publicly available sequences that were closest by sequence similarity to the outbreak cluster (n = 10) under investigation for further analyses. These were aligned with the 20 sequences from cases in our hospital and 1 sequence for each new variant in that period using MAFFT in Geneious version 11.1.4 software. Nucleic acid sequences were translated in amino acid sequences in Geneious and nonsilent mutations were analyzed and compared to the Wuhan strain and variants of concern B1.1.7, B.1.135, and P.1. The nucleic acid tree and single-nucleotide polymorphism (SNP) table were created using the ncov-tools pipeline version 1.8.0 software,^11^ using the R package ggtree. The input tree was constructed with augur tree and the default IQ-TREE method (Fig. 1).

We used transcluster software to identify transmission clusters within outbreaks based on a probabilistic approach and analyses: SNP differences, specimen collection dates, pathogen evolution rate, transmission rate, and the number of intermediate hosts between 2 samples (Supplementary Fig. 2 online). We set the molecular clock rate to 0.001 substitutions per site per year and the transmission rate as generation time of 5 days, as was previously performed.^12^ The probability that sequences were linked by ≤2 intermediate hosts was explored. Cases are clustered together when the number of transmissions is ≤2 intermediate hosts with a probability of 80%.^13^

### Statistics and ethics

Results were summarized using descriptive statistics. Research Ethics Board approval for the study was obtained from Sinai Health, Toronto, Canada (reference no. 21-0012-C).

## Description of the outbreak

**Fig. 2.**
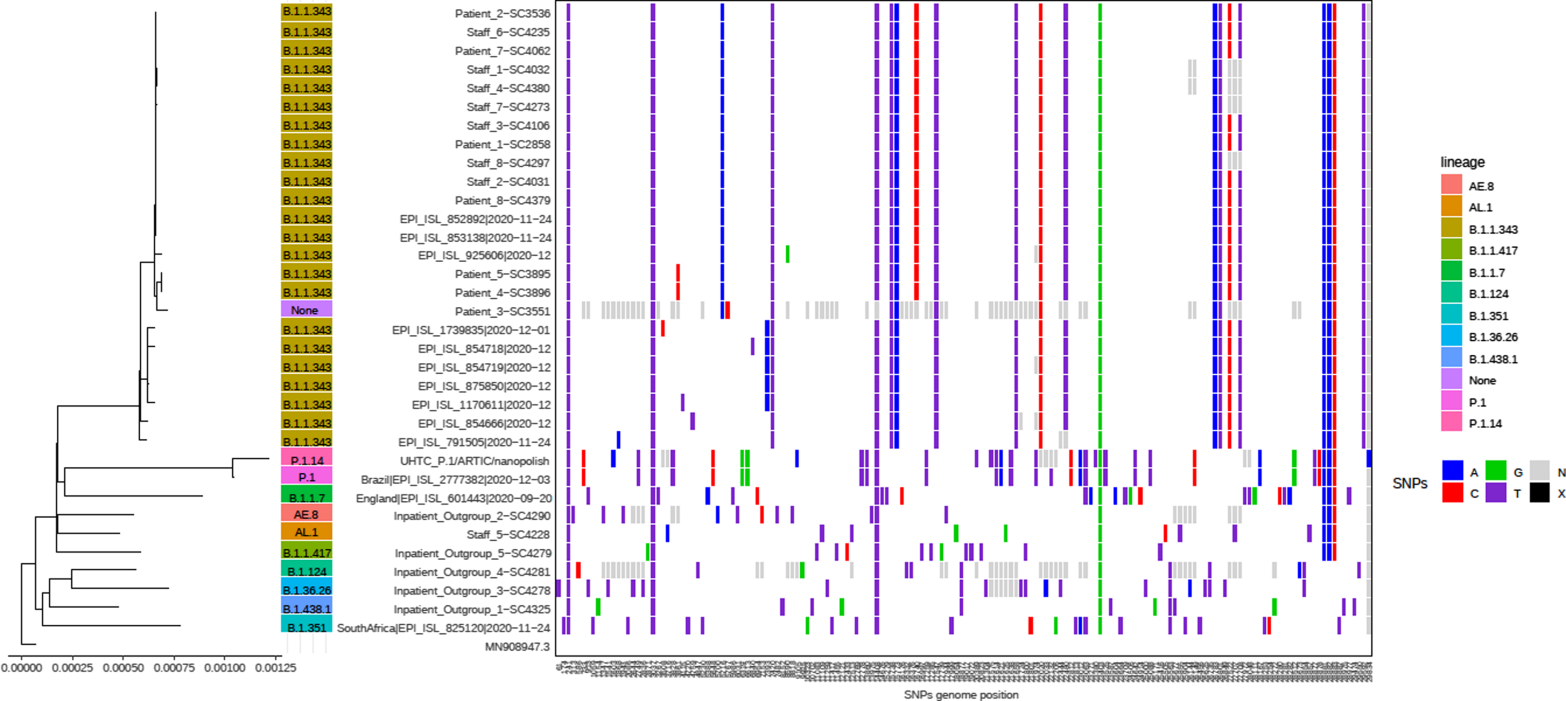
Whole-genome sequencing (WGS) phylogenetic tree and SNP table of the unit SARS-C-V-2 outbreak. Staff 5 was shown to be unrelated to the outbreak, despite presenting with symptoms during the outbreak and working extensively on the unit. All other outbreak cases sequenced were in the same phylogenetic cluster. Patient 3 (patient in geriatric chair) genome completeness was ∼60% (all other genomes included had genome completeness ≥90%) and limited the analyses but showed its inclusion in the outbreak cluster. SARS-CoV-2 WGS from 5 inpatients on other units in the hospital in the same period are indicated as outgroup and do not cluster with the outbreak showing the diversity of the circulating strains at the time. Also, 12 sequences on GISAID similar to the outbreak clusters were added.

The sentinel case (patient 1) developed symptoms on November 22, 2020, and the rRT-PCR returned positive for SARS-CoV-2 the day after (Fig. 2). We declared a suspected outbreak. All patients on the unit (Supplementary Fig. 1 online) were placed on contact–droplet precautions (ie, Canadian recommendations at the time).^14^ An initial point-prevalence survey was performed that day. No additional cases were identified.

On November 29, a repeated point-prevalence survey of all patients on the unit resulted in 2 additional cases (patients 2 and 3). Patient 2 developed symptoms on November 29, and patient 3 developed symptoms the day after. We declared an outbreak and closed the unit to admissions and nonessential visitors. All staff were tested between November 23 and 29; all 91 staff were negative and asymptomatic.

Patient 4 was transferred from a long-term care (LTC) facility where a COVID-19 outbreak occurred November 19. Patients in the unit at our hospital were maintained on contact–droplet precautions for the duration of their stay. On November 30, the patient developed symptoms and subsequently tested positive for SARS-CoV-2. A third point-prevalence survey of the unit performed on December 6 revealed 2 more asymptomatic cases (patients 5 and 6). Patient 6 developed a cough that day. Patient 7 became febrile December 8 and subsequently tested positive for SARS-CoV-2. Patient 8 developed symptoms on December 9 and tested positive.

The first staff member developed symptoms on December 8, followed by an additional staff member between December 9 and 18 (Fig. 1), and 2 additional asymptomatic staff tested positive on December 17 and 22. We closed the unit on December 22, and the unaffected patients were transferred on precautions to other units. We declared the end of the outbreak 14 days later on January 4, 2021. In total, 8 patients and 10 staff had COVID-19, and 3 patient deaths were attributed to COVID-19 (patient mortality, 37.5%). Given the timing of the outbreak, no patients were vaccinated at the time.

## Results

### Outbreak analysis and epidemiological mapping

A detailed outbreak analysis was conducted to determine the possible sources of ongoing transmission despite contact–droplet precautions on the unit. Patient 1 had been in the hospital for 10 days before developing any COVID-19 symptoms, and the source was not clear. This patient had not had any visitors, had tested negative upon admission, and had not had any known COVID-19 contacts. Patient 2 had been the roommate of patient 1, and they had interacted without masks on several occasions from November 20 to 22 prior to symptom onset in patient 1, and this was likely the source of transmission.

Review of nursing notes and interviews with nursing staff and patients revealed that patient 1 was very social on the unit, walked laps of the unit—often without a mask—and frequently interacted with other patients while asymptomatic (November 20–22). Patients 3 and 4 had advanced dementia with high fall risk and often slept in geriatric chairs at the nursing station. Both were in geriatric chairs from November 20–22 while patient 1 was walking around the unit, and they all interacted without masks.

Patient 3 also spent significant time in a geriatric chair at the nursing station, often unmasked, from November 27 to 30 while potentially contagious. Patient 5 was bedbound but required 5 medical imaging tests off the unit between November 26 and 30 and had waited for porters near patient 3 (based on documentation). Patient 6 walked unmasked around the unit and interacted with patient 3 at the nursing station on November 29. Patient 7, who developed symptoms on December 8, had shared a room with patient 6. Patient 8 also had advanced dementia, frequently slept at the nursing station in the geriatric chair, including overnight on November 28 and 29, and was unmasked and close to patient 3 during their infectious period.

Initial staff cases (staff 1–4) who had symptom onset between December 8 and 11 all worked during November 27^–^29 while patient 3 was presymptomatic close to the nursing station. During interviews, we discovered that staff members 1–4 had all had close interactions with patient 3. All staff had been masked but were not always wearing face shields, gowns, and gloves during interactions with patients while at the nursing station. An additional cluster of 5 staff cases emerged with symptom onset between December 17 and 22, potentially related to a communal food sharing event on the unit the preceding week. The case of staff member 5, whose symptom onset on December 15 did not overlap with the 2 other staff groups, was unrelated to the outbreak based on WGS (Supplementary Fig. 2).

### Whole-genome sequencing

Whole-genome sequencing (WGS) was performed to help elucidate mechanisms of transmission during the outbreak and to determine the number of introductions (Fig. 2 and Supplementary Fig. 2 online). We sequenced the SARS-CoV-2 genomes of 15 outbreak-related cases and 5 outgroup samples admitted on other hospital units as controls. The phylogenetic tree and transcluster analysis identified all suspect cases as related except for staff member 5, whose strain clustered in another clade and was unrelated to this outbreak. WGS also added precision in the outbreak investigation. Patient 4, who had initially been suspected to have acquired SARS-CoV-2 from an outbreak in an LTC facility, was subsequently attributed to our outbreak based on WGS results. Given the low evolutionary rate of SARS-CoV-2 (estimated to be 1.1×10^−3^ substitutions per site per year), it is not possible to confirm all transmission events based on WGS.^15^ The outbreak sequences were classified as B.1.1.43 and were not related to variants of concern circulating at the time.

## Discussion

We report a hospital-associated SARS-CoV-2 outbreak investigation in the pre–COVID-19 vaccine era. The outbreak involved 8 patients and 10 staff and highlights several valuable learning points. First, there is a risk of unrecognized COVID-19 acquisition in common areas on inpatient units, especially patients in geriatric chairs. These chairs with reclining positions and tray tables are commonly used on acute-care medicine units. Patients in geriatric chairs are often placed near the nursing station to further reduce the risk of falls,^16^ thereby permitting staff to tend to other duties while still monitoring the patient. Unfortunately, this leaves these patients vulnerable to interactions from other wandering patients and can lead to unrecognized high-risk interactions for the spread of COVID-19. The informal nature of interactions between patients and staff also seemed to decrease compliance with staff use of appropriate personal protective equipment (PPE), perhaps due to less access in these areas.

This outbreak also provides evidence that nosocomial acquisition can occur even in those who are bedbound in private rooms while these patients are waiting in the hallway to be taken to tests (cf, patient 5). If possible, patients should either wait in their rooms or in protected areas to decrease the risk of transmission from other residents walking around the unit.

WGS is an increasingly available tool used to support conventional outbreak investigation and management.^17,18^ In our outbreak, 1 staff member had community-acquired COVID-19, which would have been missed without WGS. Genomic analysis suggests that the outbreak was seeded by only 1 introduction. The turnaround time to obtain WGS analysis precluded us from using WGS to inform decision making during this outbreak. However, the findings from the WGS analysis provided a better understanding of transmission dynamics in this outbreak and assisted in the evaluation and future implementation of infection control measures.

Our investigation had several limitations. First, 2 of 17 outbreak-associated cases were excluded from genomic analysis because we did not have access to the specimens. As a result, it is unknown how those 2 outbreak-associated sequences compared with the result of the outbreak sequences, but we adjusted the transcluster analysis parameters correspondingly. Second, the sequence for patient 3 was of low quality due to low viral content, and one-third of the genome could not be sequenced. Nevertheless, sufficient evidence suggests that this patient was involved in this outbreak and highlights the importance of WGS in combination with traditional outbreak analysis to draw conclusions when WGS is incomplete.

Significant attention has been given to wandering residents in LTC facilities or hospitals and the role these patients could play in SARS-CoV-2 transmission, and multiple guidelines exist about strategies to mitigate this risk.^19^ This outbreak demonstrates that there is also significant risk of both acquisition and spread of SARS-CoV-2 from patients in geriatric chairs placed at the nursing station, and steps should be taken to mitigate these risks as well.

## Supplementary material

For supplementary material accompanying this paper visit https://doi.org/10.1017/ice.2023.34. click here to view supplementary material
